# Isotope Effects in Photocatalysis: An Underexplored Issue

**DOI:** 10.1021/acsomega.1c00178

**Published:** 2021-04-16

**Authors:** Carsten Günnemann, Detlef W. Bahnemann, Peter K. J. Robertson

**Affiliations:** † Institut für Technische Chemie, Leibniz Universität Hannover, Callinstraße 3, D-30167 Hannover, Germany; ‡ Laboratory “Photoactive Nanocomposite Materials”, Saint-Petersburg State University, Ulyanovskaya str. 1, Peterhof, Saint-Petersburg 198504, Russia; § School of Chemistry and Chemical Engineering, Queen’s University Belfast, Stranmillis Road, Belfast BT9 5AG, U.K.

## Abstract

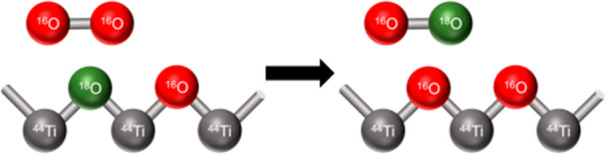

In order to improve the performance of well-established photocatalysts and to develop new potential photocatalyst materials, an understanding of the underlying mechanisms of photocatalytic reactions is of the utmost importance. An often neglected method for studying the mechanism is the investigation of isotope effects. Although just a few studies related to isotope effects exist, it has been shown to be a powerful tool for exploring mechanisms of photocatalytic processes. Most of the reports are focused on TiO_2_, which is the most studied photocatalyst, while there is a lack of data for other photocatalyst materials. This mini-review represents an overview of research utilizing isotope effects in the area of photocatalysis. The benefits and the importance of these studies will be highlighted, and the potential for these processes to be applied for the study of further photocatalytic reactions and different photocatalyst materials will be shown.

## Introduction

Semiconductor photocatalysis is a versatile technology that has been applied to a broad range of applications from treatment of contaminated water and air to energy conversion and storage.^1a−1e^ In designing and developing this process for practical commercial applications, it is critical to have a robust understanding of fundamental mechanistic processes that are occurring on the surface of the semiconductor material.^1e−1j^ A broad range of physical and chemical methods have been used in developing our understanding of these surface processes over the past four decades.^1j−1o^ One area that has not been applied to the same extent is the application of isotope effects to probe photocatalytic processes and mechanisms.

For this purpose, it is always necessary to perform two sets of experiments. In one experimental run one species (e.g., the photocatalyst) is labeled, and in a further run the same reaction is performed but with the same species unlabeled. This allows a comparison of both runs to make conclusions related to the mechanism of the investigated reaction.

The labeling of reactant molecules or the photocatalyst allows us to study the transfer of atoms between both species. For a correct interpretation of the obtained results, several points need to be considered. Besides a reaction during illumination, also a reaction in the dark needs to be performed since it might be possible that an exchange of atoms occurs spontaneously between the photocatalyst and the reactant molecules, which could lead to wrong conclusions for the reaction under illumination. Further, it is important to consider all possible reaction products that might be produced. For example, if CO_2_ is the reaction product and the incorporation of ^18^O is expected, it is necessary to detect C^16^O_2_, C^16^O^18^O, and C^18^O_2_.

In the case of the investigation of solvent isotope effects, the effect of the exchange of H_2_O by D_2_O on the rate constant is investigated. It can be recommended not only to perform experiments in pure H_2_O or D_2_O but also to consider mixtures of both solvents.

This paper explores the areas in which these techniques have been used successfully to date and also looks at the scope for more extensive application of such methods to studying the photocatalytic process promoted by semiconductor materials. In this mini-review, works from our groups as well as works from other groups are considered.

## Isotopic Labeling of Semiconductor Photocatalyst Materials and Target Molecules for Reaction

Isotopic labeling is a method to investigate the incorporation of atoms from reactant molecules into the surface of the photocatalyst and vice versa during a reaction, which allows the mechanism of chemical processes on surfaces to be followed. In the case of TiO_2_ as a photocatalyst, oxygen labeling (exchange of ^16^O by ^18^O) is commonly used. For this, two different methods exist, since the reactant molecules can be either isotopic labeled or TiO_2_ itself.

**Figure 1:**
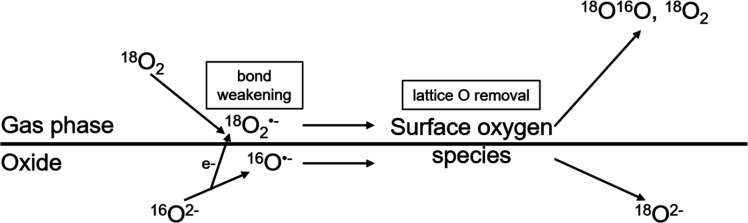
Proposed mechanism of oxygen isotopic exchange by Pichat et al.^3^ Created in analogy to ref (3).

Courbon et al.^2^ showed that after ^18^O_2_ adsorption on Ti^16^O_2_ and following UV illumination ^16^O^18^O and ^16^O_2_ can be detected in the gas phase, while the ^18^O_2_ content decreases, which proves the incorporation of ^18^O^2–^ into the surface of TiO_2_. Furthermore, they were able to confirm that only one surface oxygen anion is involved in the exchange at a time. The proposed mechanism of the oxygen isotopic exchange (OIE), as described by Pichat et al.,^3^ is depicted in Figure 1. After the excitation with light, with an energy equal to or higher than the band gap energy of TiO_2_, electron–hole pairs are formed. The conduction band electrons are able to reduce ^18^O_2_, which leads to the formation of superoxide radicals (^18^O_2_^•–^). Since the additional electron is in an antibonding orbital, the O–O bond is weakened in the superoxide radical. In the lattice of the oxide, an ^16^O^2–^ anion transfers an electron to the conduction band, forming an ^16^O^•–^ radical, which causes a weakened bond to the neighboring Ti^4+^ cations. Further, the generated species react with each other, leading to the incorporation of ^18^O^2–^ in the surface and the release of ^16^O^18^O and ^16^O_2_ to the gas phase. However, Pichat et al.^3^ pointed out that there is, to the best of their knowledge, no proof for the existence of a complex between ^18^O_2_^•–^ and ^16^O^•^^–^.

Tanaka^4^ suggested that the OIE reaction proceeds via an O_3_^•–^ radical anion, while Murata et al.^5^ were able to confirm the formation of this species. Equation 1 describes the reaction mechanism based on the formation of an O_3_^•–^ intermediate,^6^ which thus rules out a direct involvement of a superoxide species as proposed by Pichat et al.^3^ Courbon et al.^2^ found that the activity for the photooxidation of isobutane correlates with the activity for the OIE and thus concluded that an O^•–^ species is involved in both reactions, which supports the mechanism.
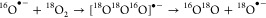
1The isotopic-labeled oxygen can be used also simultaneously with other unlabeled molecules to investigate the effect of the OIE. For example, Liao et al.^7^ reported that during UV illumination of a TiO_2_ surface no oxygen exchange between ^18^O_2_ and adsorbed CO occurs, while for adsorbed CO_2_, CO_3_, and HCOO species an oxygen exchange was observed.

**Figure 2:**
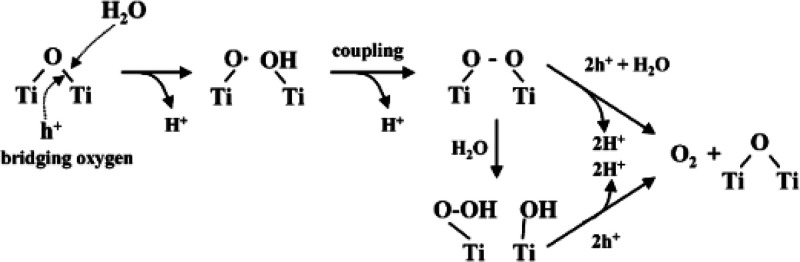
Mechanism for the oxygen evolution of TiO_2_ (rutile) in aqueous solutions with a pH between 1 and 12 during illumination. Reprinted with permission from ref (8). Copyright 2004 American Chemical Society.

A further approach is to use directly isotopic-labeled reactant molecules instead of ^18^O_2_. Isotopic-labeled water (H_2_^18^O) was used by Nakamura et al.^8^ to support their proposed mechanism (Figure 2) for the oxygen evolution of rutile in contact with aqueous solutions during illumination, with Ti–O–O–OH and Ti–O–O–Ti as intermediates. Zhang et al.^9^ showed that ^18^O-enriched cyclohexanol and benzyl alcohol form in the presence of TiO_2_ and ^16^O_2_ during illumination in benzotrifluoride cyclohexanone and benzaldehyde that contain approximately 100% ^16^O. Further, they were able to exclude the possibility of oxygen transfer from the TiO_2_ to the molecule.

Besides isotopic labeling of the reactant molecules, a further method is to label the photocatalyst. ^18^O-enriched surfaces can be prepared via different approaches. The surface of Ti^16^O_2_ can be ^18^O-enriched in contact with ^18^O_2_ at 750 K,^10^ under UV irradiation in H_2_^18^O for 12 h,^9^ or by applying a potential of 1 V in an ^18^O-containing electrolyte during illumination.^11^ It needs to be taken into account that the ^18^O-enrichment is limited to the surface of TiO_2_.^12^ Kavan et al.^13^ reported the synthesis of isotopically pure Ti^18^O_2_ by the hydrolysis of TiCl_4_ in H_2_^18^O, yielding anatase, which can be transformed to rutile by heating to 1000 °C in a vacuum.

Henderson^10^ investigated the formic acid decomposition at an ^18^O-enriched (100) TiO_2_ crystal without illumination. It was demonstrated that ^18^O-containing products are released during the decomposition (H_2_C^18^O, HC^16^O^18^OH, HC^18^O^+^, and H_2_^18^O), which confirms the incorporation of lattice oxygen into the products. Henderson^14^ also investigated the same reaction at an ^18^O-enriched (110) TiO_2_ crystal in the dark. Similar as for the (100) crystal, the transfer of lattice oxygen to the product molecules was observed. Bogdanoff and Alonso-Vante^11^ studied the photoelectrooxidation of formic acid in the presence of ^18^O-enriched TiO_2_, but no incorporation of ^18^O was observed in the detected CO_2_ molecules. Civiš et al.^15^ reported for formic acid in contact with isotopically pure Ti^18^O_2_ during illumination that no oxygen exchange occurs between the oxygen atoms of Ti^18^O_2_ and the formic acid during adsorption and decomposition since strongly bonded formate species inhibit the exchange. Although no exchange takes place, C^16^O^18^O and C^18^O_2_ can be detected. These products are formed by the spontaneous exchange of oxygen between C^16^O_2_ molecules and Ti^18^O_2_. The comparison of the studies shows that there is no direct agreement, if lattice oxygen can be incorporated in the products of the formic acid decomposition or if products containing ^18^O can be detected at all. It needs to be noted that only Civiš et al.^15^ used isotopically pure Ti^18^O_2_, while in the other reports ^18^O-enriched surfaces were investigated.

**Figure 3:**
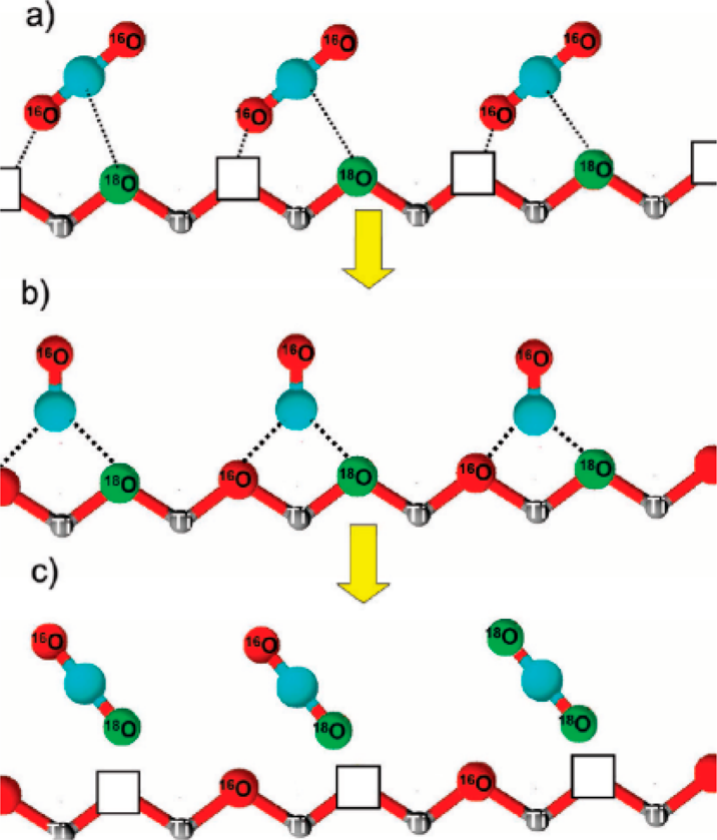
Mechanism of the spontaneous isotopic exchange between C^16^O_2_ and Ti^18^O_2_ with the involvement of oxygen vacancies. (a) Adsorption of C^16^O_2_ to the surface of Ti^18^O_2_. (b) Formation of a CO_3_ bidentate species. (c) Release of a C^16^O^18^O molecule. Reprinted with permission from ref (12). Copyright 2011 American Chemical Society.

Kavan et al.^13^ reported for the interface between isotopically pure anatase Ti^18^O_2_ and C^16^O_2_ that without illumination an OIE reaction occurs, while both C^18^O_2_ and C^16^O^18^O were detected in the gas phase. If the surface of anatase is covered with adsorbed HCl and water, no OIE was observed. Civiš et al.^12^ investigated the same interface in the dark and during illumination. As shown in Figure 3, an involvement of oxygen vacancies in the OIE reaction in the dark was found, while the whole process was found to be very fast. One oxygen atom of each C^16^O_2_ molecule coordinates to a vacancy, while the carbon atoms coordinate to lattice oxygen (Figure 3a). A CO_3_ bidentate species is formed with one oxygen atom from the C^16^O_2_ molecule being incorporated into the TiO_2_ lattice (Figure 3b). Afterward a C^16^O^18^O molecule is released from the surface, recreating an oxygen vacancy (Figure 3c). The major product of the OIE is C^18^O_2_ with a minor content of C^16^O^18^O. The adsorption of water on the surface did not suppress the OIE, and thus the water is not competing with CO_2_ for adsorption sites. By laser irradiation of the H_2_^16^O-treated Ti^18^O_2_, it was possible to enhance the OIE reaction with C^16^O_2_. Further, as products of the photocatalytic reduction of C^16^O_2_, methane and C^16^O were detected.

**Figure 4:**
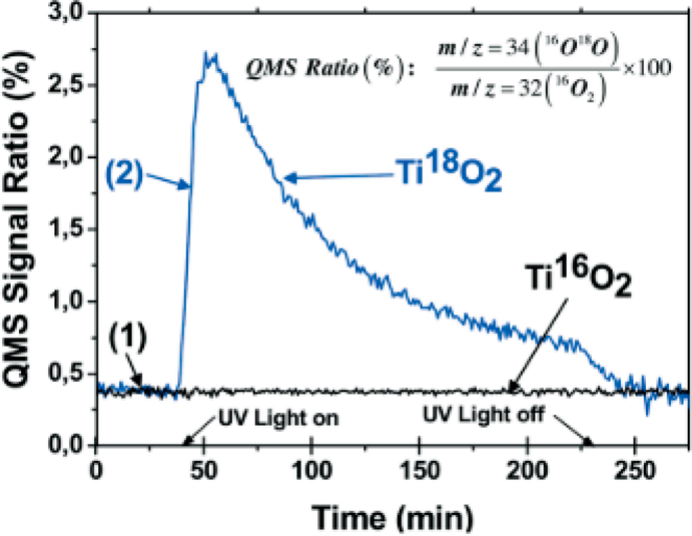
^16^O^18^O/^16^O_2_ QMS signal ratio from the photooxidation of H_2_^16^O in the presence of Ag^+^ ions with Ti^16^O_2_ and Ti^18^O_2_. Reproduced from ref (16) with permission from The Royal Society of Chemistry.

**Figure 5:**
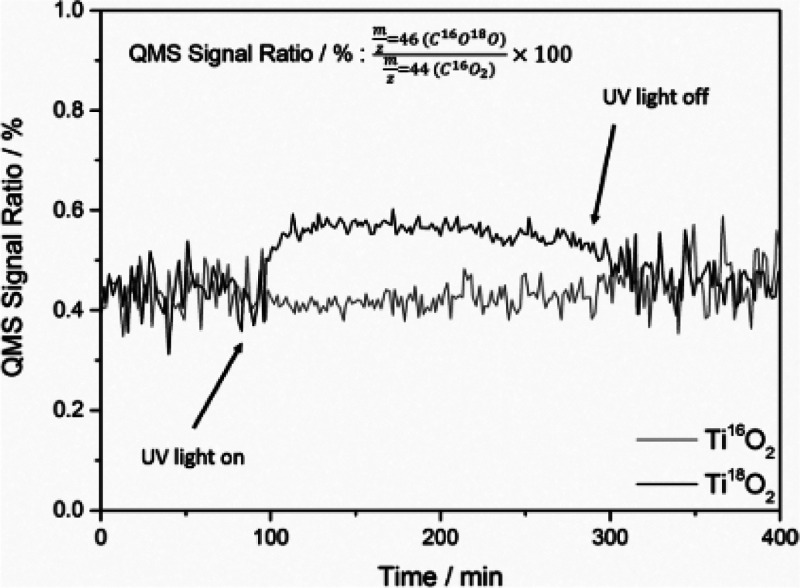
C^16^O^18^O/C^16^O_2_ QMS signal ratio from the aerobic degradation of acetaldehyde in the presence of Ti^16^O_2_ and Ti^18^O_2_ under illumination. Reprinted from ref (18). Copyright 2020, with permission from Elsevier.

**Figure 6:**
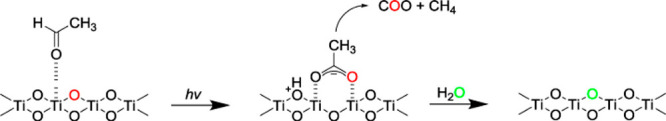
Reaction mechanism of the anaerobic degradation of acetaldehyde in the presence of TiO_2_ under illumination. Adapted from ref (18). Copyright 2020, with permission from Elsevier.

Montoya et al.^16^ investigated the H_2_^16^O photooxidation in the presence of Ti^18^O_2_ and Ag^+^ ions as electron scavengers. During illumination, a higher ^16^O^18^O/^16^O_2_ quadrupole mass spectrometry (QMS) signal ratio as compared to the dark could be detected, which turned back to the initial value after switching off the light (Figure 4). In contrast, by applying Ti^16^O_2_ instead, independent from the illumination, no change in the QMS signal ratio appeared. Since in the case of Ti^18^O_2_^16^O^18^O was evolved, it could be concluded that the photooxidation of water proceeds via a bridging oxygen from the lattice of TiO_2_, which is incorporated in the oxygen molecules. In the initial step, a 2-fold-coordinated (symbol: >) bridging oxygen (>O_br_^2–^) (eq 2) or a 2-fold-coordinated protonated bridging oxygen (>OH_br_^–^) (eq 3) reacts with a photogenerated hole, leading to the formation of a 1-fold coordinated (symbol: −) bridging oxygen radical (−O_br_^•–^) and a 1-fold coordinated bridging hydroxyl radical (−OH_br_^•^), respectively. The further steps yielding molecular oxygen according to the water redox photooxidation (WRP) mechanism are described elsewhere.^17^
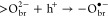
2
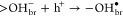
3Melchers et al.^18^ employed Ti^18^O_2_ to analyze the mechanism of the anaerobic acetaldehyde degradation during illumination. In a previous study of the same authors, the incorporation of lattice oxygen into acetate after the adsorption of acetaldehyde was proposed, which resulted in the formation of CO_2_ and CH_4_.^19^ The comparison of the C^16^O^18^O/C^16^O_2_ QMS signal ratio of Ti^16^O_2_ and Ti^18^O_2_ showed that during illumination no change for Ti^16^O_2_ occurs, while for Ti^18^O_2_ the ratio increases (Figure 5). Consequently, the incorporation of lattice oxygen into the product molecules, and thus the proposed mechanism (Figure 6), could be proven.

**Figure 7:**
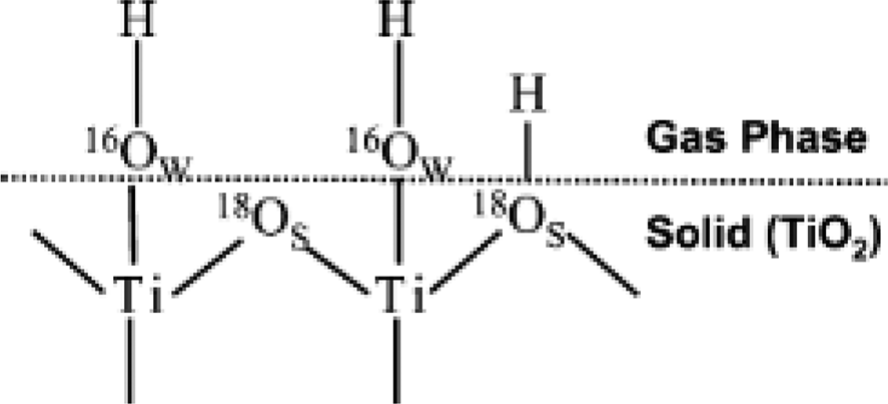
Ti^18^O_2_ surface with labeled bridging oxygens (^18^O) and unlabeled chemisorbed water species (^16^OH). Reprinted with permission from ref (20). Copyright 2013 American Chemical Society.

Montoya et al.^20^ studied the anaerobic oxidation of benzene in aqueous solutions in the presence of Ti^18^O_2_ and Ag^+^ as electron scavenger. Ti^18^O_2_ with an unlabeled hydrated surface (Figure 7), employing H_2_^16^O, was used to distinguish between two possible reaction pathways. Either hydroxyl radicals are generated from adsorbed water species (^16^OH_ads_^•^) or lattice oxygen is involved in the generation of radicals (−^18^O_br_^•–^/–^18^OH_br_^•^) and thus the oxidation of benzene. The C^16^O^18^O/C^16^O_2_ QMS signal ratio of Ti^18^O_2_ was during illumination higher compared to unlabeled Ti^16^O_2_, which indeed confirmed the involvement of bridging oxygens in the mineralization of benzene and the incorporation of these species into the product molecules.

**Figure 8:**
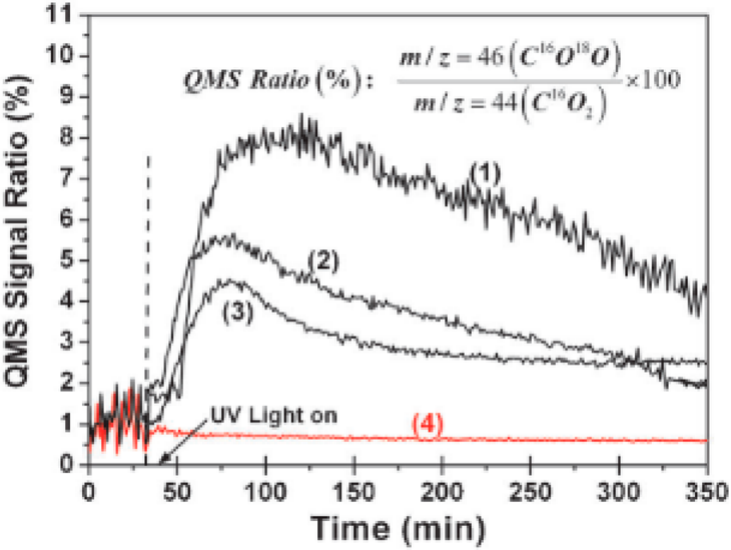
C^16^O^18^O/C^16^O_2_ QMS signal ratio from the anaerobic mineralization of benzene in acetonitrile in the presence of Ti^18^O_2_ (1–3) and Ti^16^O_2_ (4) under illumination. The following water concentrations were employed: (1) *c*(H_2_O) = 0.560 mmol L^–1^, (2) *c*(H_2_O) = 10 mmol L^–1^, (3) *c*(H_2_O) = 24 mmol L^–1^, and (4) *c*(H_2_O) = 24 mmol L^–1^. Copyright 2014 Wiley. Used with permission from ref (21).

Montoya et al.^21^ investigated also the oxidation of benzene in acetonitrile with Ti^18^O_2_ in the presence of Ag^+^ as an electron scavenger to prove the incorporation of surface oxygen of TiO_2_ into the mineralization product CO_2_. The C^16^O^18^O/C^16^O_2_ QMS signal ratio of Ti^18^O_2_ is dependent on the water concentration, while a lower concentration yields higher ratios (Figure 8). For Ti^16^O_2_ the ratio under illumination does not increase, which confirms the incorporation of surface oxygen into the CO_2_ product molecules. Based additionally on their further findings (participation of TiO_2_ terminal oxygen atoms as hole traps and the dissociative adsorption of H_2_O into terminal oxygen vacancies), the authors were able to propose a terminal-oxygen indirect electron-transfer (TOIET) mechanism. The excitation of the labeled Ti^18^O_2_ leads to the formation of free photogenerated electrons (e_f_^–^) and holes (h_f_^+^) (eq 4). A surface oxygen anion (^18^O_s_^2–^) is able to react with a free photogenerated hole, which causes the formation of a terminal radical (^18^O_s_^•–^) (eq 5). A physisorbed benzene molecule (C_6_H_6_) coordinates to a terminal radical, and an incipient covalent bond is formed (eq 6). The reaction with a further free photogenerated hole causes the formation of a phenol molecule (C_6_H_6_^18^O), which contains an ^18^O atom that originates from the surface of Ti^18^O_2_ and an oxygen vacancy (V[^18^O_s_^2–^]) (eq 7). The vacancy can be healed by the dissociative adsorption of a H_2_^16^O molecule, whereby an ^16^O_s_^2–^ anion is incorporated into the surface of Ti^18^O_2_ (eq 8). As shown in eqs 9–11, the incorporated ^16^O_s_^2–^ anion can also act as a hole scavenger, which finally results in the formation of a phenol molecule that contains an ^16^O atom (C_6_H_6_^16^O). The resulting vacancy V[^16^O_s_^2–^] is healed by the dissociative adsorption of a further H_2_^16^O molecule (eq 12). Ag^+^ ions are able to react with the free photogenerated electrons, which leads to the formation of metallic silver (eq 13). In eq 14, the complete process is summarized, which shows the exchange of an ^18^O_s_^2–^ anion with an ^16^O_s_^2–^ anion at the surface of Ti^18^O_2_.
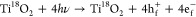
4
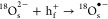
5
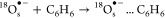
6
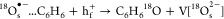
7
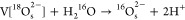
8
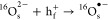
9
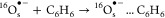
10
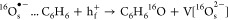
11
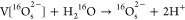
12
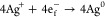
13
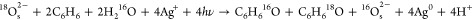
14

## Solvent Isotope Effects in Photocatalysis

Solvent isotope studies have been used extensively by organic chemists for decades. In this process, the relative rates of a reaction are compared, when they are carried out in normal water and deuterated water or “heavy” water. The solvent isotope effect (SIE) is the ratio of the rate constant in the “heavy” water (*k*_D_) to that observed in normal water (*k*_H_):
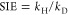
15Typically the reactions in the heavy water solvent are significantly slower, and hence the rate constants are lower than those observed in normal water. The slower reaction rate in the deuterated solvent is due to the fact that the deuterated solvent has a lower vibrational zero-point energy, and hence a greater activation energy is required to dissociate the OD bond compared to OH bonds. Consequently, the rates are slower in deuterated solvents, which are involved in reactions.

Cunningham and Srijaranai were the first to report the use of this technique for a semiconductor photocatalytic process in 1988.^22^ In their investigation of the photocatalytic degradation of isopropanol (IPA) using TiO_2_, they observed a primary solvent isotope effect of 3. It was proposed that the reduced rate of IPA destruction in D_2_O was a result of the lower quantum efficiency for the formation of OD^•^ radicals on the TiO_2_ surface in the heavy water solvent. As a result of this, there would be a lower number of OD^•^ radicals on the TiO_2_ surface, which would be available to attack the isopropanol. On the basis of this proposal, they suggested that the photogeneration of hydroxyl radicals was the rate-determining step for the photocatalytic process.

Robertson et al. also observed a solvent isotope effect of 3 for the photocatalytic destruction of the cyanobacterial toxin microcystin-LR using a P25 TiO_2_ photocatalyst.^23a^ The solvent isotope effect observed by Cunningham for the decomposition of IPA was the same as that reported by Robertson for the cyanotoxin, considering the substantial difference in structure and the molecular mass of the two substrates. Robertson suggested that this was also a confirmation of Cunningham and Srijaranai’s proposal that the hydroxyl radical generation on the photocatalyst surface was also the rate-determining step for the photocatalytic reaction.

In a subsequent study, Robertson and co-workers investigated the solvent isotope effect on the degradation of microcystin-LR (MC-LR) and another cyanobacterial chemical metabolite, geosmin (GSM), using a Hombikat K01/C TiO_2_ photocatalyst.^23b^ In this case a solvent isotope effect of 1.5 was observed for microcystin and geosmin,^23b^ which was approximately 50% lower than that found in the previous studies by Robertson et al. and Cunningham and Srijaranai (Table 1).

**Table 1:** Kinetic Isotope Effect Based on the Photocatalytic Destruction of GSM and MC-LR in Normal and Heavy Water with Hombikat K01/C TiO_2_ as a Photocatalyst^a^

GSM			MC-LR	
solvent	*k* (μM min^–1^)	relative rate	*k* (μM min^–1^)	relative rate
H_2_O	1.56	1.0	8.55	1.0
D_2_O	0.97	0.62	5.44	0.64

a Reprinted from ref (23b). Copyright 2011, with permission from Elsevier.

In this study, Robertson et al. proposed that the solvent isotope effect observed for both molecules was mediated via hydroxyl radicals, generated from the subsequent reduction of the superoxide radical anion, produced at the conduction band. After being generated, the superoxide would be hydrated or deuterated by the solvent to form a hydroperoxide ion (eq 16). The hydroperoxide ions may then interact to form hydrogen peroxide, which would then generate OH^•^ (or OD^•^) radicals following an electron transfer from the conduction band again. This may be rate determining since O_2_ has to be generated at the conduction band prior to the interaction with the solvent and the subsequent formation of OD^•^ or OH^•^ species (eqs 17 and 18).
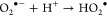
16
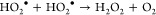
17
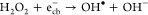
18It was suggested that the observed solvent isotope effect could be a result of the rate of the reaction of the solvent with superoxide species rather than by the rate of reaction of OH^•^ (or OD^•^) on the microcystin or geosmin. If the isotope reaction depended on this latter reaction, one would expect it to be the same no matter what photocatalyst or species being oxidized was utilized.

An interesting observation is the fact that the solvent isotope effect is approximately 3 for P-25 and approximately 1.5 for K01/C. They also suggested that since similar kinetic solvent isotope effects were observed for different substrate molecules on the same photocatalyst materials the interaction of the solvent with the photocatalyst and the rate of oxidation of the solvent were probably the rate-determining steps for the photocatalytic reaction, as opposed to conduction band reduction of oxygen as previously proposed by Gerischer and Heller.^24^ Furthermore, Robertson et al. proposed that the reason the kinetic solvent isotope effect observed in this subsequent work was smaller than that in their previous study and Cunningham’s work was due to the fact that different photocatalyst materials were employed,^23b^ and hence the effect was likely to be dependent on the photocatalyst material.

**Figure 9:**
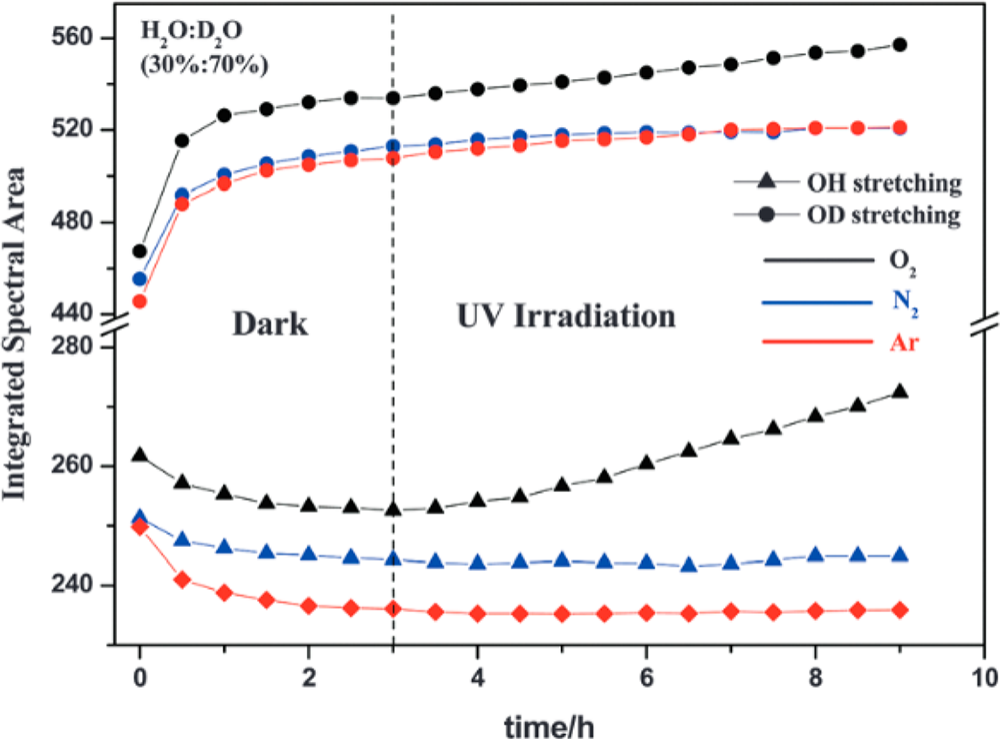
Evolution of the intensity of the integrated spectral areas of OH and OD stretching groups before and after UV irradiation: effect of dissolved O_2_, N_2_, and Ar on the adsorption of H_2_O–D_2_O on the TiO_2_ surface. Reprinted from ref (25a). Published by the PCCP Owner Societies.

Belhadj et al. used solvent isotope effects to study the adsorption of water and deuterium oxide on TiO_2_ surfaces in the dark and under UV(A) irradiation using in situ ATR-FTIR spectroscopy under aerobic and anaerobic conditions.^25a^ Under dark conditions in a mixture of H_2_O and D_2_O solvents, an isotopic exchange was found to occur on the surface of the TiO_2_ material. Following irradiation with UV(A) light, the quantity of both OH and OD groups was found to be increasing in the presence of molecular oxygen. Additionally, hydroperoxide was generated through a photocatalytic process under aerobic conditions, which was believed to be produced as a result of the reduction of molecular oxygen adsorbed at the TiO_2_ surface by the photogenerated conduction band electrons, as opposed to being generated via water oxidation from valence band holes. It was also demonstrated from the spectroscopic studies that under conditions where the percentage of H_2_O was significantly less than that of D_2_O there was an exchange of solvent groups on the TiO_2_ surface with the OD^–^ ions, having a stronger adsorption affinity to the photocatalyst compared to the OH^–^ ions. Following illumination with UV light, both OH and OD groups were generated on the photocatalyst surface in the presence of oxygen. The generation of these groups also increased the hydrophilicity of the TiO_2_ surface. If the experiment was conducted under either a nitrogen or argon atmosphere, there was no evidence of the formation of OH and OD groups, and the hydrophilicity was inhibited (Figure 9). This result indicated that under UV irradiation oxygen played a critical role in both the photocatalytic response and the photoinduced hydrophilicity.

**Figure 10:**
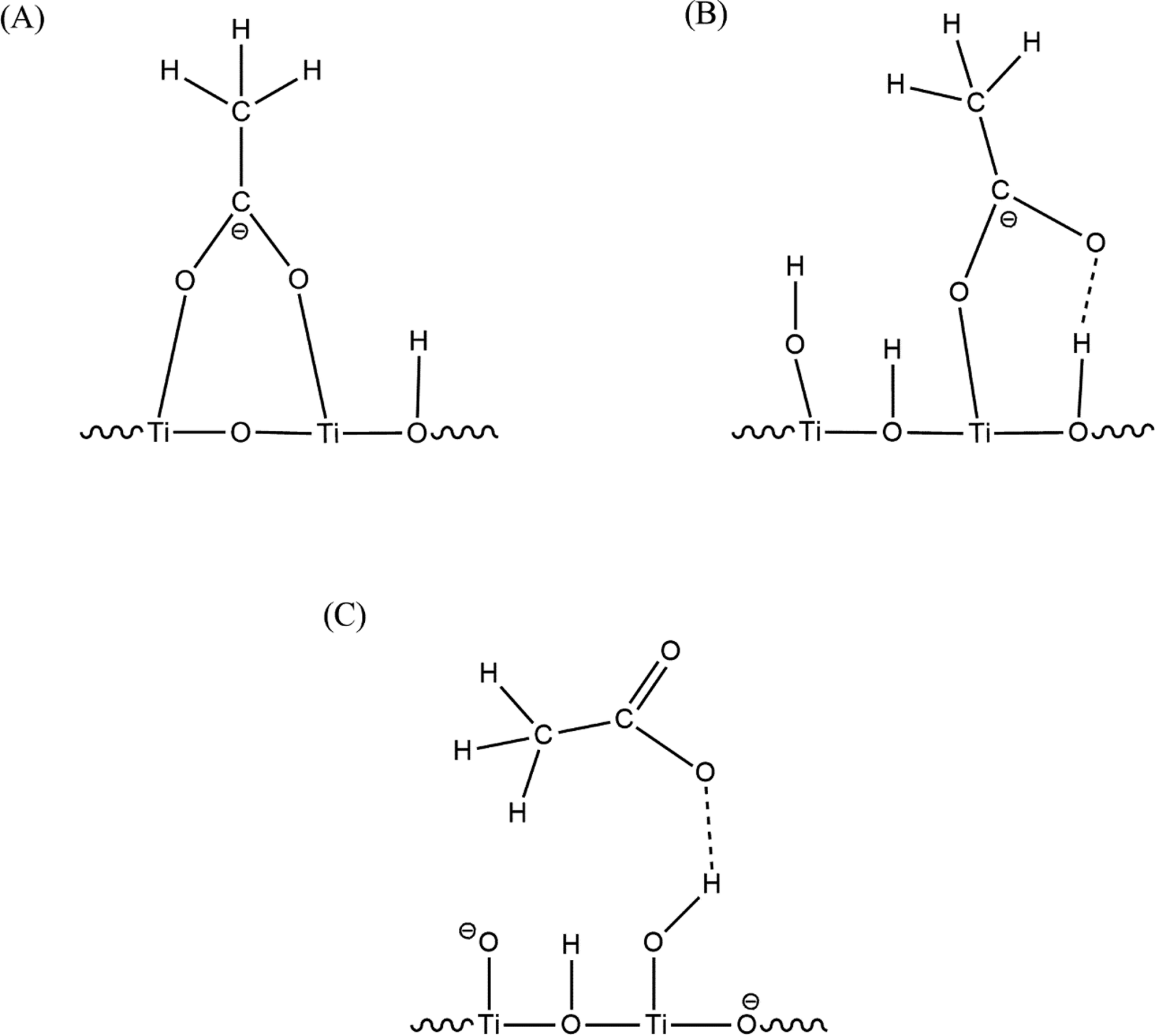
Schematic representation for the adsorption of acetate on the anatase surface (UV100) in the dark at pH < pH_zpc_ (A), pH ≈ pH_zpc_ (B), pH > pH_zpc_ (C). Reprinted from ref (25b). Copyright 2016, with permission from Elsevier.

In a subsequent study, the adsorption and photocatalytic degradation of acetate on TiO_2_ surfaces was investigated in H_2_O and D_2_O by both attenuated total reflection Fourier transformed infrared spectroscopy (ATR-FTIR) and EPR spectroscopy.^25b^ Different interactions between the adsorbed acetate and OD groups resulted from the isotopic exchange on the TiO_2_ surface following adsorption of D_2_O. The interaction of the acetate with the TiO_2_ surface was found to be strongly influenced by the pH, and a range of surface complexes with the acetate were observed to form. Under acidic conditions, the formation of a bidentate structure involving two distinct Ti atoms appeared to be the preferred complex structure. At pH values close to the point of zero charge for the TiO_2_, the acetate favored a monodentate complex, formatted through adsorption to the positively charged TiO_2_ anatase material (Figure 10).

**Figure 11:**
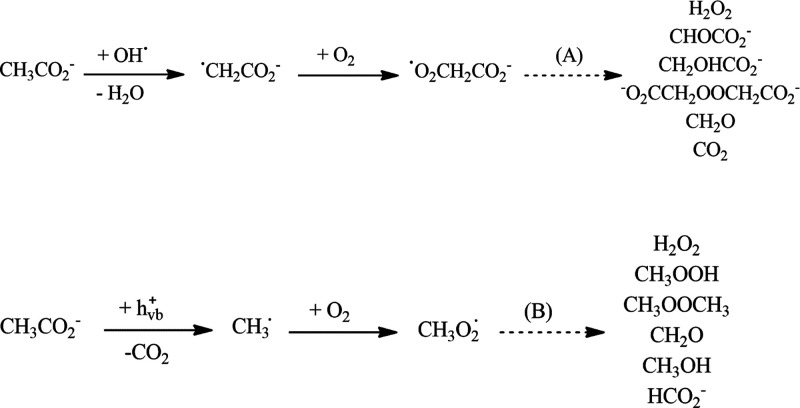
Proposed mechanism for the photocatalytic reaction of acetate at pH 9 (A) and pH 3 (B). Reprinted from ref (25b). Copyright 2016, with permission from Elsevier.

Following irradiation with UV(A) light, hydroxyl radicals were observed under alkaline conditions, while methoxy radicals were generated under acidic conditions. Two different degradation pathways were proposed for the acetate under acidic and alkaline conditions (Figure 11), which were supported by the experimental studies performed using ATR-FTIR and EPR spectroscopy. Overall, the results of the EPR study suggested that under alkaline conditions acetate degradation was mainly promoted by attack by valence band generated hydroxyl radicals. Under acidic conditions, the degradation appeared to occur via direct oxidation via photogenerated valence band holes.

Solvent isotope studies were also used for the investigation of the simultaneous photocatalytic degradation of formaldehyde and hydrogen evolution on a platinized TiO_2_ material under an oxygen-free atmosphere.^25c^ Using QMS and ATR-FTIR spectroscopy for analysis, the main reaction products obtained from the photocatalytic degradation of 20% formaldehyde were hydrogen and carbon dioxide in a ratio of 2 to 1. From the solvent isotope study, it was found that the rate of mineralization of formaldehyde to CO_2_ is significantly reduced with increasing concentration of D_2_O. Following the investigation of the solvent isotope effect on the system using ATR-FTIR analysis, it was proposed that the formaldehyde oxidation was promoted by attack by OD^•^ radicals, formed from the reaction with the photogenerated valence band hole. This reaction generated a surface-adsorbed deuterated formic acid (HCOOD), which subsequently underwent further oxidation by valence band holes in a photo-Kolbe-type reaction. The photogenerated conduction band electrons were proposed to simultaneously reduce H^+^ and D^+^, originating from both formaldehyde and D_2_O, to form molecular HD. The yield of the HD gas was found to be strongly influenced by the solvent and was maximized when the ratio of H_2_O:D_2_O was 20%:80%. The proposed mechanism for the simultaneous hydrogen production and formaldehyde oxidation in the presence of D_2_O is summarized in eqs 19–26 below:^25c^
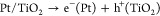
19
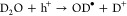
20
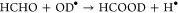
21
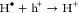
22
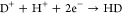
23
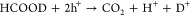
24
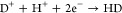
25
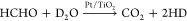
26

## Scope for Research Development and Focus

Each of the studies considered in this paper related to the labeling of photocatalyst materials and target molecules have utilized TiO_2_ as the photocatalyst. This is also the case for the studies dealing with solvent isotope effects. Although TiO_2_ is the most studied photocatalyst material, there has only been a relatively small number of reports related to isotope effects. As has been detailed above, the use of these isotope studies has enabled important insights to be gained for photocatalytic reactions on TiO_2_, but there is still significant scope for further studies using these techniques.

For future research, while also investigating the isotope effects of the photocatalytic decomposition of other substrates on TiO_2_ materials, it would be important to extend the studies to other photocatalyst materials as well. For example, Fe_2_O_3_, WO_3_, CdS, C_3_N_4_, and SrTiO_3_ are known as photocatalytically active materials, while the labeling of the catalyst or reactant molecules would allow us to get deeper insights into the corresponding mechanisms using these materials.

With respect to TiO_2_ itself, there are many more reaction mechanisms that should be investigated based on solvent isotope effects. In particular, the involvement of terminal hydroxyl or oxygen radicals in the photocatalytic mechanism, as one of the initial steps, might be either established or refuted.

The technique could also be used for kinetic studies, particularly in the case of rapidly decomposing intermediates, which may be more easily followed in the heavy water solvent.

## Conclusion

The investigation of isotope effects represents a powerful tool in the area of photocatalysis to study the mechanisms of the reactions occurring on the surface of photocatalyst materials. Using this technique, it is possible to elucidate whether photocatalyst surface atoms are transferred into product molecules, while also the incorporation of atoms from reactant molecules into the surface of the photocatalyst can be observed. Furthermore, by exchanging H_2_O by D_2_O, the ratio of the rate constants between both solvents can be followed to investigate whether the generation of hydroxyl radicals is the rate-determining step of a reaction. Using D_2_O as a solvent has a further advantage since it allows the determination of whether hydrogen atoms in product molecules originate from reactant molecules or from the solvent. Consequently, it is important to perform such studies to allow the determination of the mechanistic pathway of the photocatalytic process. It should be noted that isotope effect studies are, however, not enough as the sole process to provide such detail, but in combination with other tools, they can provide important information on such processes.

In conclusion, it is clear that the application of isotope studies is a versatile and useful tool for studying photocatalytic reactions; however, the technique has been only applied in a relatively small number of investigations. There is therefore great scope for the further application of this technique in the field of semiconductor photocatalysis, and it is anticipated that this will be an area of growing interest within the photocatalytic research community over the next few years.

## References

[ref1] aHoffmann M. R.; Martin S. T.; Choi W.; Bahnemann D. W. Environmental Applications of Semiconductor Photocatalysis. Chem. Rev. 1995, 95, 69–96. 10.1021/cr00033a004.

[ref2] Courbon H.; Formenti M.; Pichat P. Study of Oxygen Isotopic Exchange over Ultraviolet Irradiated Anatase Samples and Comparison with the Photooxidation of Isobutane into Acetone. J. Phys. Chem. 1977, 81, 550–554. 10.1021/j100521a012.

[ref3] Pichat P.; Courbon H.; Enriquez R.; Tan T. T. Y.; Amal R. Light-Induced Isotopic Exchange between O_2_ and Semiconductor Oxides, a Characterization Method That Deserves Not to Be Overlooked. Res. Chem. Intermed. 2007, 33, 239–250. 10.1163/156856707779238667.

[ref4] Tanaka K. Intermediate of Oxygen Exchange Reaction over Illuminated Titanium Dioxide. J. Phys. Chem. 1974, 78, 555–556. 10.1021/j100598a019.

[ref5] Murata C.; Hattori T.; Yoshida H. Electrophilic Property of O_3_^–^ Photoformed on Isolated Ti Species in Silica Promoting Alkene Epoxidation. J. Catal. 2005, 231, 292–299. 10.1016/j.jcat.2005.01.012.

[ref6] Mikhaylov R. V.; Lisachenko A. A.; Titov V. V. Investigation of Photostimulated Oxygen Isotope Exchange on TiO_2_ Degussa P25 Surface upon UV–Vis Irradiation. J. Phys. Chem. C 2012, 116, 23332–23341. 10.1021/jp305652p.

[ref7] Liao L. F.; Lien C. F.; Shieh D. L.; Chen M. T.; Lin J. L. FTIR Study of Adsorption and Photoassisted Oxygen Isotopic Exchange of Carbon Monoxide, Carbon Dioxide, Carbonate, and Formate on TiO_2_. J. Phys. Chem. B 2002, 106, 11240–11245. 10.1021/jp0211988.

[ref8] Nakamura R.; Nakato Y. Primary Intermediates of Oxygen Photoevolution Reaction on TiO_2_ (Rutile) Particles, Revealed by in Situ FTIR Absorption and Photoluminescence Measurements. J. Am. Chem. Soc. 2004, 126, 1290–1298. 10.1021/ja0388764. 14746503

[ref9] Zhang M.; Wang Q.; Chen C.; Zang L.; Ma W.; Zhao J. Oxygen Atom Transfer in the Photocatalytic Oxidation of Alcohols by TiO_2_: Oxygen Isotope Studies. Angew. Chem. 2009, 121, 6197–6200. 10.1002/ange.200900322. 19343745

[ref10] Henderson M. A. Formic Acid Decomposition on the {110}-Microfaceted Surface of TiO_2_(100): Insights Derived from ^18^O-Labeling Studies. J. Phys. Chem. 1995, 99, 15253–15261. 10.1021/j100041a048.

[ref11] Bogdanoff P.; Alonso-Vante N. A Kinetic Approach of Competitive Photoelectrooxidation of HCOOH and H_2_O on TiO_2_ Anatase Thin Layers via on-Line Mass Detection. J. Electroanal. Chem. 1994, 379, 415–421. 10.1016/0022-0728(94)87165-5.

[ref12] Civiš S.; Ferus M.; Kubát P.; Zukalová M.; Kavan L. Oxygen-Isotope Exchange between CO_2_ and Solid Ti^18^O_2_. J. Phys. Chem. C 2011, 115, 11156–11162. 10.1021/jp201935e.

[ref13] Kavan L.; Zukalova M.; Ferus M.; Kürti J.; Koltai J.; Civiš S. Oxygen-Isotope Labeled Titania: Ti^18^O_2_. Phys. Chem. Chem. Phys. 2011, 13, 11583–11586. 10.1039/c1cp20775j. 21597637

[ref14] Henderson M. A. Complexity in the Decomposition of Formic Acid on the TiO_2_(110) Surface. J. Phys. Chem. B 1997, 101, 221–229. 10.1021/jp961494i.

[ref15] Civiš S.; Ferus M.; Zukalová M.; Kubát P.; Kavan L. Photochemistry and Gas-Phase FTIR Spectroscopy of Formic Acid Interaction with Anatase Ti^18^O_2_ Nanopartciles. J. Phys. Chem. C 2012, 116, 11200–11205. 10.1021/jp303011a.

[ref16] Montoya J. F.; Bahnemann D. W.; Salvador P.; Peral J. Catalytic Role of Bridging Oxygens in TiO_2_ Liquid Phase Photocatalytic Reactions: Analysis of H_2_^16^O Photooxidation on Labeled Ti^18^O_2_. Catal. Sci. Technol. 2017, 7, 902–910. 10.1039/C6CY02457B.

[ref17] Salvador P. Mechanisms of Water Photooxidation at N-TiO_2_ Rutile Single Crystal Oriented Electrodes under UV Illumination in Competition with Photocorrosion. Prog. Surf. Sci. 2011, 86, 41–58. 10.1016/j.progsurf.2010.10.002.

[ref18] Melchers S.; Schneider J.; Bahnemann D. W. Isotopic Studies on the Degradation of Acetaldehyde on Anatase Surfaces. Catal. Today 2020, 340, 318–322. 10.1016/j.cattod.2018.10.016.

[ref19] Melchers S.; Schneider J.; Emeline A. V.; Bahnemann D. W. Effect of H_2_O and O_2_ on the Adsorption and Degradation of Acetaldehyde on Anatase Surfaces—An In Situ ATR-FTIR Study. Catalysts 2018, 8, 417 10.3390/catal8100417.

[ref20] Montoya J. F.; Ivanova I.; Dillert R.; Bahnemann D. W.; Salvador P.; Peral J. Catalytic Role of Surface Oxygens in TiO_2_ Photooxidation Reactions: Aqueous Benzene Photooxidation with Ti^18^O_2_ under Anaerobic Conditions. J. Phys. Chem. Lett. 2013, 4, 1415–1422. 10.1021/jz400580b. 26282293

[ref21] Montoya J. F.; Bahnemann D. W.; Peral J.; Salvador P. Catalytic Role of TiO_2_ Terminal Oxygen Atoms in Liquid-Phase Photocatalytic Reactions: Oxidation of Aromatic Compounds in Anhydrous Acetonitrile. ChemPhysChem 2014, 15, 2311–2320. 10.1002/cphc.201402043. 24827557

[ref22] Cunningham J.; Srijaranai S. Isotope-Effect Evidence for Hydroxyl Radical Involvement in Alcohol Photo-Oxidation Sensitized by TiO_2_ in Aqueous Suspension. J. Photochem. Photobiol., A 1988, 43, 329–335. 10.1016/1010-6030(88)80029-7.

[ref23] aRobertson P. K. J.; Lawton L. A.; Cornish B. J. P. A.; Jaspars M. Processes Influencing the Destruction of Microcystin-LR by TiO_2_ Photocatalysis. J. Photochem. Photobiol., A 1998, 116, 215–219. 10.1016/S1010-6030(98)00312-8.

[ref24] Gerischer H.; Heller A. The Role of Oxygen in Photooxidation of Organic Molecules on Semiconductor Particles. J. Phys. Chem. 1991, 95, 5261–5267. 10.1021/j100166a063.

[ref25] aBelhadj H.; Hakki A.; Robertson P. K. J.; Bahnemann D. W. In Situ ATR-FTIR Study of H_2_O and D_2_O Adsorption on TiO_2_ under UV Irradiation. Phys. Chem. Chem. Phys. 2015, 17, 22940–22946. 10.1039/C5CP03947A. 26266701

